# Young People’s Experiences and Perceptions of YouTuber-Produced Health Content: Implications for Health Promotion

**DOI:** 10.1177/1090198120974964

**Published:** 2020-11-27

**Authors:** Jane Harris, Amanda Atkinson, Michael Mink, Lorna Porcellato

**Affiliations:** 1 Liverpool John Moores University, Liverpool, UK; 2 California State University Sacramento, CA, USA

**Keywords:** adolescents, digital health, health communication, health promotion, social media, YouTube

## Abstract

The growing diversity and uptake of social media has increased the sources of health information available to young people. YouTube is one of the most popular social media platforms for young people in the United Kingdom, and YouTubers are the most important influencers on the platform producing diverse health content. They are increasingly recognized by popular media and public health organizations as a potentially influential source of health information for young people. This study aimed to qualitatively explore young people’s experiences and perceptions of YouTuber health content. Focus groups (November 2017 to January 2018) with 85 young people (13–18 years) were recruited from schools in a single county in North West England. The findings suggest young people’s engagement with YouTuber health content is dependent on how they encounter it, YouTubers’ motivations for producing it, and the perceived relatability, sincerity, and generalization present in this content. The study confirms YouTuber health content was one of the many sources of health information used by young people and was most frequently encountered during young people’s routine viewing. Collaboration between public health organizations and YouTubers could be promising in communicating health messages to young people already engaged with these YouTubers, as part of wider campaigns or interventions. These messages could be particularly effective if they focused on experiences and norms rather than advice, remained consistent with YouTubers’ existing health content, incorporate clear indicators of accuracy into their narrative, and state their intention to benefit young people.

Social media is one of the multiple sources of health information available to young people in the digital age. U.K. survey data report 93% of 16 to 24 year olds have used social media in the past 12 months (Office for National Statistics, 2019) and 69% of 12 to 15 year olds have a social media profile (Ofcom, 2019). Channel complementary theory (Dutta-Bergman, 2004) recognizes that young people’s use of multiple health information sources is influenced by the function each source serves, namely, access to medical expertise, tailorability, convenience, and anonymity (Ruppel & Rains, 2012). In a large-scale focus group study of adolescents’ online health seeking, Gray et al. (2005a) identified three issues that affected participants’ recall and perception of online health information: (1) previous experience of the symptoms and source, (2) saliency of the information, and (3) the credibility of the source based on expertise, trustworthiness, and empathy. This suggests popular, routinely used social media sites could play a role in young people’s health information seeking.

The video sharing site YouTube is extremely popular among young people. According to U.K. survey data, 93% of 16 to 24 year olds (Office for National Statistics, 2019) and 89% of 12 to 15 year olds (Ofcom, 2019) used YouTube in 2019. YouTube has been described as an intermediary participatory culture where both commercial organizations and amateurs produce content side-by-side and so are constantly negotiating and co-constructing their relationships with their viewers and each other (Burgess & Green, 2018). These characteristics have led to the rise of the YouTube celebrity (Gamson, 2011; Marwick, 2015b) or “YouTuber” who upload both topic-based videos and video blogs (vlogs) of their daily lives to a large audience (e.g., British comedy, music and gaming YouTuber KSI, 21 million subscribers). YouTubers are among the internet celebrities referred to as “influencers.” Abidin (2018) defines influencers as branded, vocational online stars who use highly engaging content to maintain a large social media following. U.K. press reporting contains numerous examples of YouTubers providing commentary on health issues and sharing their health behaviors with a large audience of predominantly teenagers and young adults (Burgess & Green, 2018; Sheffield, 2014).

Coates et al. (2019) are among the few researchers that have attempted to demonstrate the impact of YouTuber health content on young people’s health behaviors. In a randomized controlled trial design (176 children, 9–11 years) they found children exposed to influencers showing unhealthy snacks had a significantly increased overall calorie intake and unhealthy snack intake compared with those who viewed nonfood products. In a follow-up qualitative study, they found children (10–11 years) felt affected by the accessibility and familiarity of YouTubers unhealthy food marketing, but children also believed they were able to resist it (Coates et al., 2020). Despite only sparse evidence, it has been proposed that YouTubers could be a particularly relatable source of health information for young people (Beer, 2008; Uhls & Greenfield, 2012). This is illustrated by Public Health England’s decision to use YouTubers to communicate messages about topics such as risk, body image and relationships in “Rise Above,” its most recent PHSE program (Public Health England, 2017) but evaluation of this program has not been published.

This research has therefore evolved from a significant gap in the literature as little research has focused on qualitatively exploring young people’s experiences and perceptions of YouTuber health content. These insights are valuable because U.K. health campaigns (Public Health England, 2017) are beginning to make use of YouTubers in health improvement, and these activities should be informed by the perspectives of young people. By developing a thorough understanding of the circumstances in which young people engage with YouTuber health content (2011), this study provides important initial data on the contexts in which health improvement efforts are most likely to be effective.

## Materials and Methods

The study aim was to explore young people’s (age 13–18 years) perceptions and experiences of YouTuber-produced health content. The study used an interpretivist, exploratory qualitative approach. Seven focus groups were undertaken with 85 young people (13–18 years) from a convenience sample from three secondary schools and sixth-form colleges in North West England. Three focus groups were stratified by age (according to school year group) and gender for participants under 16 years (Gibson, 2007; Heath et al., 2009; Porcellato et al., 2002). Focus groups for young people aged 16 years and over (*n* = 4) were larger and mixed gender as it was logistically easier for the college to recruit whole tutor groups to participate.

Ethical approval was granted by Liverpool John Moores University Research Ethics Committee (Ref: 17/PBH/017). A teacher from each school acted as primary gatekeeper, granting the researcher access to the students in their care. Teachers signed a gatekeeper consent form and assisted the researcher in identifying participants from their classes. With regard to the composition of focus groups, preestablished friendship or peer groups were used to establish rapport, ease of discussion, and reflect the peer context in which health-related social norms are constructed (Eder & Fingerson, 2001; Gibson, 2007). The researcher had no prior relationship with any school staff or pupils. All participants received an information sheet and gave written consent prior to the focus group. Opt-out parental consent was given for young people under 16 years (see Supplemental Appendix 2). The lead researcher (JH, female, PhD student in public health) conducted the focus groups between November 2017 and January 2018 in a private room, on school premises, during normal school hours.

A topic guide was developed from the literature and refined after the first focus groups. Open-ended questions asked participants about health information on YouTube, and YouTubers and their health content (Supplemental Appendix 1). A broad approach to health was taken (Spencer, 2014), which included physical and mental health, risk behaviors, relationships, and confidence, based on a questionnaire with young people (*n* = 931), which was an earlier part of this mixed methods study (Harris, 2019). The focus groups began with an icebreaker (Gibson, 2007) where the groups twice ranked sources of health information according to frequency of use and accuracy. Photographs of five popular YouTubers and four 2- to 3-minute excerpts from YouTuber videos were used to prompt the discussion. Three videos were YouTuber experiences of mental health conditions (one male, one female) and physical activity (male). The fourth video had been produced by a YouTuber for Public Health England’s “Rise Above” PHSE campaign (Public Health England, 2016), focusing on social norms of health risk behaviors.

Focus groups (lasting 25–60 minutes) were transcribed verbatim by JH and analyzed in Nvivo version 11. Braun and Clarke’s (2006) stages of familiarization, coding, producing, reviewing, and labelling themes were followed iteratively from descriptive to interpretative analysis. By the eighth focus group, no new codes were identified, and it was concluded data saturation had been reached (Guest et al., 2016). Analyses were conducted by JH, codes were reviewed by LP (female, PhD, reader in public health), and themes reviewed by LP, AA (female, PhD, senior researcher in public health), and MM (male, PhD, professor in public health) to ensure reliability. The Consolidated Criteria for Reporting Qualitative Research (COREQ) were followed (Supplemental Appendix 2).

## Results

Seven focus groups were undertaken with 85 young people aged 13 to 18 years (mean age = 15.6, mode = 17; Table 1). Just over half of the sample (53%) were female. Shorter verbatim quotes have been embedded within the narrative, and six longer, illustrative excerpts (referenced in the text) have been included in Table 2. Five themes were identified: (1) young people’s method for finding YouTuber health content, (2) the relatability, (3) sincerity, (4) generalization present in this content, and (5) YouTubers’ perceived motivations for producing health content.

**Table 1.** Focus Group (FG) Participants.

Focus group	Number of participants	Gender	Year group	Age	School type
FG1	5	Male	9	13–14 years	Single-sex, state funded
FG2	9	Male	10	14–15 years	Mixed, studio school^a^
FG3	7	Female	10	14–15 years	Mixed, studio school
FG4	15	Mixed	12	16–17 years	Mixed, sixth-form college^b^
FG5	15	Mixed	12	16–17 years	Mixed, sixth-form college
FG6	16	Mixed	13	17–18 years	Mixed, sixth-form college
FG7	18	Mixed	13	17–18 years	Mixed, sixth-form college

a A studio school is a type of state funded free school in the United Kingdom. Studio schools accept pupils aged 14 to 19 years, are smaller than traditional high schools (<300 pupils), and combine traditional academic and vocational courses through links with local business. ^b^A sixth-form college is an educational institution in the United Kingdom where pupils aged 16 to 19 years study for advanced school-level qualifications.

**Table 2.** Extended Quotations From Focus Groups.

Excerpt 1	R: Ok, do we want to have a look at [YouTuber photograph]. Anyone aware of any content she makes? *F5: Mental health* *F6: She makes mental health videos about her anxiety* (FG6, 17–18 years)
Excerpt 2	*M1: If you have like a proper mainstream YouTuber right and you watch them every single day and then one day it pops up with like a video about mental health. Cause I know like [YouTuber] did that . . . a change of like you know the pace. It actually like makes you think about it . . . because it’s something that you know your favorite YouTuber is watching and thinking about*. (14 years, Male, FG2)
Excerpt 3	*M1: Cos if you look on the internet for it, it’s good but then like you can’t actually see—* *M2: You can’t see a real person* *M1: The person saying it. . . . Because anyone could have just said it. But if its someone on YouTube said it then you know they do mean it.* *M3: You know they’re gonna kind of be right as well, because if they’re going through that same thing, they’re gonna obviously have done their research as well. . . . What they’re saying is most likely gonna be true.* (13–14 years, Males, FG1)
Excerpt 4	*If they hear someone like [YouTuber] saying oh I’ve got anxiety, they could think well I want to have anxiety. It’s kind of like turning it into . . . if [YouTuber]’s got it, then it’s ok to have it so I will have it*. (F5, 17 years, FG6)
Excerpt 5	*M1: You’ve got some people who kind of like push mental health because they’ve seen something from their own fan base . . . say a fan goes to the YouTuber and says, “Aw, I’m feeling depressed, your videos helped me” and that’s kind of like the spark. . . . It’s the realisation for a lot of YouTubers . . . how like influence [sic] they have over people and that their videos are actually helping people in a sense. So . . . they need to continue this and it kind of affects them. . . . Whereas others, they see it as aww yeah, if I talk about mental health, I could get sponsored by this, I could get sponsored by that. Look how much money I’m gonna get. It’s being fake for you know like public reputation.* (14 years, Male, FG2)
Excerpt 6	*M7: I was watching this video about like weight loss and stuff and then half way through they mention this diet pill that you can take and I was like ah no, I’ll just get off it do you know what I mean. You can’t trust any of it.* (17 years, FG7)

### Method of Finding YouTuber Health Content

Young people discussed two distinct methods of encountering YouTuber produced health content: (1) health information they had actively searched for and (2) health information encountered during their routine viewing of YouTubers. Participants of all ages and genders gave examples of health content encountered in videos of YouTubers they viewed regularly. Participants were shown photographs of YouTubers who produced mental health content and at least one participant in every focus group was aware of this content without prompting (Table 2, Excerpt 1).

Older participants (16 years and over) said while they were aware of this health content, it was not their main motivation for watching YouTubers: “He’s talked a bit about depression in the past but I don’t go to him to find that out . . . from just watching his videos” (M7, 17 years, FG7). In contrast, younger participants would search for health information on YouTube to gauge the seriousness of their concern. A 14-year-old female participant described how “they have like physiotherapy stuff on YouTube because when I sprained me ankle, I used it . . . to stretch your ankle to repair the ligaments faster” (F4, 14 years, FG3). When she was asked how she judged the quality of this information, she replied, “’cos, I went the doctor . . . and he was like it looks perfect, you did it perfectly. And the ligaments were Ok after a week instead of a month” (F4, 14 years, FG3). This suggests that for younger teens, YouTube information could be a stopgap or sense-check before seeking advice from a healthcare professional.

Younger males (under 16 years) said they would prioritize content from a familiar YouTuber when searching for health information: “If there’s someone I watch has done a video on what I’m looking for . . . I’d go to them first” (M3, 13 years, FG1). The appeal appeared to be convenience “because you don’t have to ask anyone directly, you can just search it online on YouTube” (M1, 13 years, FG1) and reluctance to discuss certain health issues such as mental health, “You still have that like huge thing that you do not want to talk about it. . . . So you’d go to YouTube to be like, I need something, to figure out like what you need” (M1, 14 years, FG2). This suggests YouTubers are one of the sources of health information available to young people but engagement may be influenced by age and gender.

### The Advantages of Relatability

Participants across all ages and genders used the term *relatable* to describe how YouTubers portrayed an everyday version of health events similar to their own experiences. Characteristics of *relatable* YouTuber health content were (1) YouTubers sharing emotional aspects of their experiences was “. . . more supportive. It’s not quite got any sort of medical twist to it, it’s more like something you can relate to . . . watch it and say well I feel like that” (F4, 17 years, FG6). (2) YouTubers were perceived as honest about their experiences. “She doesn’t make it look good or nothing. She genuinely says it’s not like a nice thing to have. . . . I feel like that’s why she’s more relatable to people with anxiety, like she’ll speak openly about it” (F1, 14 years, FG3). (3) YouTubers talking about health “looked friendly, they didn’t look too serious . . . like a normal conversation you would have with a friend . . . it kind of makes you feel chill with them . . . these people are like my friends” (M1, 14 years, FG2) or “more like a big sister than anything” (F2, 14 years, FG3). This suggests some young people viewed YouTubers as an extension of peer-constructed norms, with evidence suggesting young people are more likely to emulate the health behaviors of admired associates (Prinstein & Dodge, 2008).

Participants said finding a YouTuber with similar health issues “can help you . . . if you’re suffering in the same ways” (M1, 13 years, FG1). The youngest male group gave the example of “somebody with depression might watch that video and think that they’re not the only one” (M3, 13 years, FG1). Participants described how YouTubers relatable health experiences could raise awareness of health topics they had not previously considered. A female participant specified how a male YouTuber could assist discussion of mental health among her male peers “because . . . he’s showing that males suffer with mental health problems too and it’s not a bad thing. It doesn’t make them any less of a person” (F4, 17 years, FG6). A 14-year-old male gave the example of a YouTuber they regularly watched prompting them to think about mental health issues for the first time (excerpt 2). This suggests YouTubers could be promising in addressing stigma associated with certain health issues and help-seeking among young people.

### Sincerity

The perceived sincerity of YouTubers’ health content also influenced young people’s engagement. Older participants’ primary concern was YouTubers not placing themselves in a position of professional expertise but being “open and truthful . . . about your experience and this is what it’s like . . . learn from the experiences” (F4, 17 years, FG7). Younger participants’ judgements about sincerity were more complex. The 14-year-old male group described simple editing styles as more sincere, “It’s . . . videos that go slow . . . non-edited, camera on you, straight to the point. You wouldn’t have like flashes and explosions just like Woah! Depression. Boom” (M6, 14 years, FG2). Some felt ongoing engagement with YouTubers helped them assess sincerity. They recalled changes in the YouTubers’ health behavior observed through regular viewing; for example, mental health, “You know he’s suffered from it and he said he’s had it six months before he realized” (M1, 14 years, FG2), or improved fitness, “Because he wasn’t skinny at first and he . . . wasn’t as good as he is now” (F2, 14 years, FG3). In other cases, participants’ reasoning was less explicit and included subtle aspects such as YouTubers’ tone, “You can kind of tell by the way they speak . . . the sincerity of it” (M1, 14 years, FG2). This suggests younger viewers’ feelings of trust and familiarity potentially limited their ability to appraise the accuracy of YouTuber health content (Excerpt 3).

### Generalization

Participants expressed concern that YouTubers could generalize their own health experiences by providing a biased account, unrepresentative of the diversity existing among their viewers. Older participants discussed a male YouTuber video on depression where they felt he did not present alternative treatment options, “They might need to talk about that and then they will be ok. Whereas I feel like he was just trying to go with the medication” (F5, 17 years, FG7) and hadn’t “highlighted the negatives either, like all the change in dosages over six months, all the trial and error” (F8, 17 years, FG6). Participants across all ages expressed concern that YouTubers’ discussion of anxiety and depression could “romanticize” (F4, 14 years, FG3) the conditions for younger viewers, potentially leading to self-diagnosis to emulate the YouTuber in question (Excerpt 4).

### YouTuber Motivations for Producing Health Content

YouTubers’ motivations for producing health content, specifically their scope for personal gain, influenced whether young people responded positively or negatively. A 14-year-old male distinguished between YouTubers making mental health content to genuinely help viewers (viewed positively) and those motivated by viewing figures and profits (viewed negatively, Excerpt 5). A 14-year-old female described how advice from “your mum or your grandparents . . . you feel like they’re just trying to control you . . . ’cos they love you and ’cos they are your family,” whereas a YouTuber producing health content was viewed as less biased (“Well they don’t know me, but they’re still telling me not to do it because it’s unsafe. And you believe it more”; F1, 14 years, FG3). This suggests young people’s engagement is influenced by the YouTubers’ perceived motivations for producing health content. For some young people this was strongly linked to the presence of commercial sponsorship.

Participants generally felt confident identifying commercial sponsorship or advertising in YouTuber content through verbal disclosure by the YouTuber, “#ad” appearing in the video title and written disclosure in the video description. Participants of all ages were aware “there’s a law now . . . that you can’t do an ad unless you specify that you have” (M1, 13 years, FG1), referring to the UK Advertising Standards Agency rules requiring paid-for social media advertising to be obviously identifiable (Advertising Standards Agency, 2020). Participants felt advertising could sometimes “sneak in” (F7, 17 years, FG7) to YouTuber videos, identifiable by excessive, consistent positivity about a product and a more scripted tone: “The way they’re acting . . . better toward it, which they must have told them to do really” (M1, 13 years, FG1).

Participants’ reaction to commercial sponsorship varied by age. Younger participants were critical of YouTubers who “sold out” (M2, 14 years, FG2) by endorsing brands and placing profits before their audience. These younger viewers tended to identify with an online community associated with YouTubers where “actual YouTubers just care about making videos and like the community” (M1, 14 years, FG2). Commercial influences present in YouTuber health content effected their perceptions of relatability, sincerity, and likelihood of engagement.

Some participants questioned YouTubers’ motives to make sponsored health content, feeling YouTubers may present false health experiences to sell a product (e.g., weight-loss products, Excerpt 6). Generally, older participants were less troubled by commercial influences in YouTuber health content. They felt YouTubers have “got to get their money somewhere. They’re not just doing it out of the niceness of their heart are they” (F8, 17 years, FG7) and that “as long as it’s a good video I don’t mind” (F7, 17 years, FG7).

## Discussion

This study is among the first to qualitatively explore young people’s perceptions and experiences of YouTuber health content. Five themes influenced how young people engage with YouTuber health content: their method of finding this content, the relatability, sincerity and generalization present, and YouTubers’ perceived motivations for producing health content. This study confirms YouTubers are among the many sources of health information young people encounter in the digital age, indicating their potential for communicating health messages as part of young people’s health improvement. The five themes identified provide insight into which aspects of young people’s engagement with YouTuber health content could be particularly promising.

Young people were more likely to encounter YouTuber health information during routine viewing rather than active searching. This distinction between actively seeking and passively absorbing health information (Gray et al., 2005b) is often referred to as instrumental versus noninstrumental use (Papacharissi & Rubin, 2000). Health-related mass media campaigns are a widely used example of passive health information that are known to be more successful when exposure occurs during routine media use (Wakefield et al., 2010). This suggests YouTubers could be particularly effective communicating health messages to young people who are already regular viewers. Working collaboratively with YouTubers who are already producing content on the required health topic could assist health improvement efforts to reach an audience of young people already predisposed toward these health messages.

Some younger male participants prioritized content produced by a familiar YouTuber when searching for health information. This is promising considering the acknowledged difficulties in accessing traditional health services among this group due to gendered norms of masculinity and health seeking behaviors (Booth et al., 2004; Gray et al., 2005a). As discussed, channel complementary theory (Dutta-Bergman, 2004) recognizes that young people’s use of multiple health information sources is influenced by the function each source serves, namely, access to medical expertise, tailorability, convenience, and anonymity (Ruppel & Rains, 2012). Our findings suggest younger male participants were aware YouTuber health information lacked medical expertise but found it convenient due to the immediate and anonymous way they could access tailorable, low-cost information (Best et al., 2014; Gray et al., 2005b). YouTubers could therefore be promising for communicating health messages to young males.

YouTuber health content was appealing to young people when it was “relatable” to participants’ own health experiences and perceived to be sincere. This agrees with Gray et al. (2005a), where three issues affected participants’ recall and perception of health information: (1) previous experience of symptoms and source, (2) saliency of the information, and (3) credibility of the source based on expertise, trustworthiness, and empathy. Several participants said YouTubers were “like a big sister” and “like my friends” suggesting they were akin to a personal source. Personal sources of health information are important during adolescence; when peer influence increases while parental influence wanes (Gray et al., 2005b; Inchley et al., 2017). YouTubers may be viewed by some young people as an extension of peer norms (Lim, 2013), with young people more likely to emulate the health behaviors of those they admire (Prinstein & Dodge, 2008). Research suggests celebrities can similarly influence the health information individuals retain due to their perceived credibility, and audience conditioning to react positively to their advice (Hoffman & Tan, 2015). This suggests YouTubers may be promising in communicating positive norms of health behavior to young people. Participants also felt YouTuber content could help raise awareness and reduce stigma toward certain health issues. Research has shown anticipated and perceived stigma is an important barrier to help-seeking (Clement et al., 2015), suggesting YouTubers could encourage young people to seek health advice from professional sources.

However, the link between YouTuber influence and young people’s admiration (Prinstein & Dodge, 2008) was dependent on the perceived sincerity of their health content. Judgements about YouTuber sincerity were affected by a combination of rapport with the YouTuber, the YouTubers’ motivations for creating content and perceived concern about their audience. The importance of sincerity appeared to wane as young people matured. Younger male viewers were critical of what Cocker and Cronin (2017) define as YouTubers’ shift from “charismatic authority” to the “routine of charisma.” The participants described this as “selling out” or “going mainstream,” producing less personal and more generic, commercialized content for personal gain (“routinization of charisma”) rather than audience benefit (“charismatic authority”; Cocker & Cronin, 2017; Glatt, 2017). Research outlines how YouTubers use highly competent self-branding techniques to build trust (Abidin, 2018; Khamis et al., 2017) and give their audience a sense of custodianship over the YouTubers’ channel (Cocker & Cronin, 2017). This suggests health improvement efforts involving YouTubers are more likely to be effective among young people who have developed this level of trust and familiarity with YouTubers (Abidin, 2018; Marwick, 2015a). However, health improvement collaborations with YouTubers must maintain consistency with YouTubers’ previous health messages, tone, and persona (Abidin, 2018; García-Rapp, 2017) to be well received by their young audience. Public health organizations should approach YouTubers whose previous content is consistent with the health messages being communicated and clearly state the intended audience benefits, otherwise they risk alienating the young people they target.

Some participants expressed concerns that YouTubers generalizing their personal experiences could encourage self-diagnosis or lead to alternative treatments being overlooked. Concerns about generalization, sincerity, and commercial sponsorship suggest opportunities to increase young people’s digital health literacy toward YouTubers. Younger participants’ feelings of trust and familiarity could sometimes limit their ability to appraise YouTuber health content accuracy. Previous research on the credibility of online health information reports young people base judgements on a combination of expertise, trustworthiness, and empathy (Gray et al., 2005a). Health improvement collaborations which encourage YouTubers to explicitly identify indicators of accuracy in their narrative (e.g., citing sources, background information on collaborators) could thus have an educative effect for younger teenagers. Older participants appeared less invested in these familiar and trusting relationships but more accepting of sponsorship and not particularly troubled if the YouTuber content was also entertaining. As van Dijck (2013) claims, the neoliberal and commercialized infrastructures with underlie social media content can become for young people “an infrastructure they do not question” (p. 175). Using Personal Social and Health Education (PSHE) to equip young people with skills to critique the underlying ideology and ecology of social media platforms could allow young people across the 13 to 18 age group to make informed decisions about the YouTuber health information they encounter.

This study suggests YouTubers could have a promising role in young people’s health improvement. However, we acknowledge that health professionals may be cautious about using YouTubers for health improvement, as the easy sharing and spreading of this content could lead them to lose control of how their health messages are received (McKee et al., 2018) In addition, media-based health improvement efforts enter a crowded environment and compete with multiple factors including commercial marketing, social norms, entrenched behaviors, and wider structural and environmental factors impacting upon young people’s health. YouTuber interventions are therefore most likely to be effective as part of wider interventions that link young people to other components such as service provision or product distribution (Michie et al., 2011; Randolph & Viswanath, 2004; Wakefield et al., 2010).

## Limitations

Focus group recruitment used convenience sampling. While the sample achieved maximum variation with older, younger, female, and male young people represented (Emmel, 2013), the focus groups were recruited from a single county in North West England and so the findings cannot be generalized to other populations. For logistic reasons, focus groups 16 years and over were larger (>12 participants) and mixed gender. Mixed gender groups may present some limitations as male participants can tend to speak more frequently, thus reducing the opportunities for females to participate (Krueger & Casey, 2015). However, for young people in older age groups, homogeneity of gender is not always essential and both single and mixed gender focus groups can be successful (Gibson, 2007) as illustrated in our study where 62% of the mixed gender discussion was by female participants. Larger groups can limit all young people from participating, although in this study all participants in the larger groups contributed to the discussion, which may have been aided by using preestablished groups. All focus groups recruited young people from preestablished groups to ease rapport and discussion; however, using peer groups could emphasize existing power imbalances and tensions among peers. While these imbalances did not appear to hinder the group, there may have been underlying tensions which the researcher was unaware of. In school environments practicalities and flexibility often have to be prioritized over an ideal sample (Gibson, 2007).

## Conclusions

The study aim was to explore young people’s (age 13–18 years) perceptions and experiences of YouTuber produced health content. The findings confirm YouTubers are one of the many online and offline sources of health information used by young people. YouTuber health content was most frequently encountered during young people’s routine viewing and particularly appealing to younger males due to convenience and confidentiality. The findings suggest collaboration between public health organizations and YouTubers could be promising in communicating health messages to young people as part of wider campaigns or interventions. These messages could be particularly effective if they focused on experiences and norms, remained consistent to experiences in YouTubers’ existing content, incorporate clear indicators of accuracy into their narrative, and state their intention to benefit young people. The findings suggest younger teenagers who are already actively engaged with YouTubers might be more predisposed to receive YouTuber health messages. There is also some evidence to suggest that YouTubers may be an effective way to encourage young males in help seeking and this issue warrants further research.

## Supplemental Material

sj-docx-1-heb-10.1177_1090198120974964 – Supplemental material for Young People’s Experiences and Perceptions of YouTuber-Produced Health Content: Implications for Health Promotion Click here for additional data file. Supplemental material, sj-docx-1-heb-10.1177_1090198120974964 for Young People’s Experiences and Perceptions of YouTuber-Produced Health Content: Implications for Health Promotion by Jane Harris, Amanda Atkinson, Michael Mink and Lorna Porcellato in Health Education & Behavior

sj-docx-2-heb-10.1177_1090198120974964 – Supplemental material for Young People’s Experiences and Perceptions of YouTuber-Produced Health Content: Implications for Health Promotion Click here for additional data file. Supplemental material, sj-docx-2-heb-10.1177_1090198120974964 for Young People’s Experiences and Perceptions of YouTuber-Produced Health Content: Implications for Health Promotion by Jane Harris, Amanda Atkinson, Michael Mink and Lorna Porcellato in Health Education & Behavior
