# Supplementary material for: Young People’s Experiences and Perceptions of YouTuber-Produced Health Content: Implications for Health Promotion
Source: Health Educ Behav. 2020 Nov 27;48(2):199–207. doi: 10.1177/1090198120974964 (PMC7961622; doi:10.1177/1090198120974964)
Supplement: sj-docx-1-heb-10.1177_1090198120974964 – Supplemental material for Young People’s Experiences and Perceptions of YouTuber-Produced Health Content: Implications for Health Promotion [file sj-docx-1-heb-10.1177_1090198120974964.docx]

**Appendix 1: Focus group topic guide**

1. **Ice breaker – ranking exercise**

Participants will be asked to rank sources of health information. The researcher will provide the group with ten pre-printed cards with sources of health information: GP/doctor, NHS Direct, Parents/guardians, Other family, Friends, YouTube, Social Media, Newspaper/Magazines, TV, Websites. Spare cards will be provided so that participants can add any other sources of health information they feel are missing.

The participants will then be asked to reach a group consensus in order rank the sources of health information by laying them out on the table in order. They will be ask to do this in two ways:

1) Where do you look for health information most often?

2) Which health information is the best quality and most accurate?

The researcher will encourage participants to discuss their decision making process and use prompts throughout the exercise.

1. **YouTube and health**

One of the sources of information we looked at was YouTube:

1. What kind of health information can you find on YouTube?
   *Prompts: Source, messages, channels, who is intended to watch them. Experience vs factual*
2. What are the advantages/ good points of this information on YouTube?
   *Prompts: Easy to access? Private? Easy to understand? Easy to relate to?*
3. What are the disadvantages/ problems with health information on YouTube?

*Prompts: is it accurate, opinion vs fact, quality, bias*

1. Do you think in general YouTube is a good or bad place to look for health information

*Why. Do you have any examples?*

**3) YouTubers and Health**

Moving on to think about YouTubers,

1. Can you think of examples of YouTubers talking about health messages?
2. What are the advantages/ disadvantages of this information?
   *Prompts: relatable, easy to understand, easy to access, quality, truthfulness, accuracy*
3. Here are five pictures of popular UK YouTubers (*taken from netnographic study)*, can you tell me what kind of health messages these YouTubers talk about?

**4) YouTube videos**

The participants will be shown three short exerts (1-3 minutes) of YouTube videos selected from the netnographic analysis. Videos will be selected to ensure they are subjects which are suitable for the focus group age and which do not contain any potentially distressing or sensitive topics.

**Video 1: Attitudes – a clip will be selected in which a YouTuber is giving their opinion on a certain health related behaviour**

1. What was the main message of this video?
2. After watching this video, what is your opinion of behaviour x? – *Has it changed your opinions in anyway?*
3. Was this video good quality? – why/why not?
4. Was the information in this video true/accurate? – why/why not?

**Video 2: Subjective norms – a clip will be selected in which a YouTuber is talking about their experiences of a certain healthy/unhealthy behaviour**

1. What is the main message of this video?
2. Do you think health behaviour x is important/ not important? *How do you feel about people who do behaviour x?*
3. Was this video good quality? – why/why not?
4. Was the information in this video true/accurate? – why/why not?

**Video 3: Perceived behavioural control – a clip will be shown in which a YouTuber is performing a certain healthy/unhealthy behaviour**

1. What is the main message of this video?
2. After watching this video, do you think behaviour x is something you could do? *How confident do you feel about it? Why do you feel this way?*
3. Was this video good quality? – why/why not?
4. Was the information in this video true/accurate? – why/why not?

**5) Closing questions**

1. After watching these videos, how do you feel about the health topics YouTubers talk about? Has your opinion changed in anyway?
2. Do you have anything you would like to add?
3. Are there any questions about what we have discussed
