# Supplementary material for: Young People’s Experiences and Perceptions of YouTuber-Produced Health Content: Implications for Health Promotion
Source: Health Educ Behav. 2020 Nov 27;48(2):199–207. doi: 10.1177/1090198120974964 (PMC7961622; doi:10.1177/1090198120974964)
Supplement: sj-docx-2-heb-10.1177_1090198120974964 – Supplemental material for Young People’s Experiences and Perceptions of YouTuber-Produced Health Content: Implications for Health Promotion [file sj-docx-2-heb-10.1177_1090198120974964.docx]

**Appendix 2: COREQ: 32-item checklist**

| **No** | **Item** | **Guide questions/description** |  |  |
| --- | --- | --- | --- | --- |
|  | | **Domain 1: Research team and reflexivity** | | |
|  | | ***Personal Characteristics*** | | |
| 1. | Interviewer/facilitator | Which author/s conducted the interview or focus group? | Stated | See 'Materials and Methods' section. |
| 2. | Credentials | What were the researcher's credentials? *E.g. PhD, MD* | Stated | See 'Materials and Methods' section. |
| 3. | Occupation | What was their occupation at the time of the study? | Stated | See 'Materials and Methods' section.. |
| 4. | Gender | Was the researcher male or female? | Stated | See 'Materials and Methods' section. |
| 5. | Experience and training | What experience or training did the researcher have? | Stated | See 'Materials and Methods' section. |
|  | | ***Relationship with participants*** | | |
| 6. | Relationship established | Was a relationship established prior to study commencement? | Stated | Schools were self-selecting. The researcher had no previous relationship with the teachers and participants before the programme of research began but the schools did participate in an earlier questionnaire study. |
| 7. | Participant knowledge of the interviewer | What did the participants know about the researcher? e*.g. personal goals, reasons for doing the research* | Stated | The researcher introduced themselves and their reasons for doing the research at the beginning of each focus group. |
| 8. | Interviewer characteristics | What characteristics were reported about the interviewer/facilitator? e.g. *Bias, assumptions, reasons and interests in the research topic* | Stated | See ‘Materials and Methods’ section. |
|  | | **Domain 2: study design** | | |
|  | | ***Theoretical framework*** | | |
| 9. | Methodological orientation and Theory | What methodological orientation was stated to underpin the study? *e.g. grounded theory, discourse analysis, ethnography, phenomenology, content analysis* | Stated | See ‘Materials and Methods’ section. |
|  | | ***Participant selection*** | | |
| 10. | Sampling | How were participants selected? *e.g. purposive, convenience, consecutive, snowball* | Stated | See ‘Materials and Methods’ section. |
| 11. | Method of approach | How were participants approached? e*.g. face-to-face, telephone, mail, email* | Stated | See ‘Materials and Methods’ section. |
| 12. | Sample size | How many participants were in the study? | Stated | See ‘Materials and Methods’ section. |
| 13. | Non-participation | How many people refused to participate or dropped out? Reasons? | Stated | As existing peer-groups were initially approached to participate by class teachers, it is not possible to say how many participants refused to participate. One female participant (aged 14 years) chose to leave the focus group before it was completed and her data was not included. |
|  | | ***Setting*** | | |
| 14. | Setting of data collection | Where was the data collected? e*.g. home, clinic, workplace* | Stated | See ‘Materials and Methods’ section. |
| 15. | Presence of non-participants | Was anyone else present besides the participants and researchers? | Stated | See ‘Materials and Methods’ section. |
| 16. | Description of sample | What are the important characteristics of the sample? *e.g. demographic data, date* | Stated | See Table 1. |
|  | | ***Data collection*** | | |
| 17. | Interview guide | Were questions, prompts, guides provided by the authors? Was it pilot tested? | Stated | See ‘Materials and Methods’ section and Appendix 1 |
| 18. | Repeat interviews | Were repeat interviews carried out? If yes, how many? | Stated | No. |
| 19. | Audio/visual recording | Did the research use audio or visual recording to collect the data? | Stated | See ‘Materials and Methods’ section. Focus groups were audio recorded |
| 20. | Field notes | Were field notes made during and/or after the interview or focus group? | Stated | See ‘Materials and Methods’ section. Recordings were transcribed verbatim. Throughout the data collection and analysis, a reflective diary was used to allow the researchers to examine their roles and biases. |
| 21. | Duration | What was the duration of the interviews or focus group? | Stated | See ‘Materials and Methods’ section. |
| 22. | Data saturation | Was data saturation discussed? | Stated | See ‘Materials and Methods’ section. |
| 23. | Transcripts returned | Were transcripts returned to participants for comment and/or correction? | Stated | Transcripts were not returned to the participants after the focus groups. |
|  | | **Domain 3: analysis and findings** | | |
|  | | ***Data analysis*** | | |
| 24. | Number of data coders | How many data coders coded the data? | Stated | See ‘Materials and Methods’ section. |
| 25. | Description of the coding tree | Did authors provide a description of the coding tree? | Stated | See ‘Materials and Methods’ section. |
| 26. | Derivation of themes | Were themes identified in advance or derived from the data? | Stated | See ‘Materials and Methods’ section. |
| 27. | Software | What software, if applicable, was used to manage the data? | Stated | See ‘Materials and Methods’ section. |
| 28. | Participant checking | Did participants provide feedback on the findings? | Stated | Participants did not provide feedback on the findings. |
|  | | ***Reporting*** | | |
| 29. | Quotations presented | Were participant quotations presented to illustrate the themes / findings? Was each quotation identified? e*.g. participant number* | Stated | See 'Findings' section |
| 30. | Data and findings consistent | Was there consistency between the data presented and the findings? | Stated | See 'Findings' section |
| 31. | Clarity of major themes | Were major themes clearly presented in the findings? | Stated | See 'Findings' section |
| 32. | Clarity of minor themes | Is there a description of diverse cases or discussion of minor themes? | Stated | See 'Findings' section |
